# Transmission of monkeypox/mpox virus: A narrative review of environmental, viral, host, and population factors in relation to the 2022 international outbreak

**DOI:** 10.1002/jmv.28534

**Published:** 2023-02-06

**Authors:** Daniel Pan, Joshua Nazareth, Shirley Sze, Christopher A. Martin, Jonathan Decker, Eve Fletcher, T. Déirdre Hollingsworth, Michael R. Barer, Manish Pareek, Julian W. Tang

**Affiliations:** ^1^ Department of Respiratory Sciences University of Leicester Leicester UK; ^2^ Department of Infectious Diseases and HIV Medicine University Hospitals of Leicester NHS Trust Leicester UK; ^3^ Li Ka Shing Centre for Health Information and Discovery, Big Data Institute University of Oxford Oxford UK; ^4^ NIHR Leicester Biomedical Research Centre Liecester UK; ^5^ Department of Cardiovascular Sciences University of Leicester Leicester UK; ^6^ Department of Clinical Microbiology University Hospitals of Leicester NHS Trust Leicester UK; ^7^ Department of Virology University Hospitals of Leicester NHS Trust Leicester UK

**Keywords:** monkeypox, mpox, sexual contact, transmission, viral load

## Abstract

Monkeypox virus (MPXV) has spread globally. Emerging studies have now provided evidence regarding MPXV transmission, that can inform rational evidence‐based policies and reduce misinformation on this topic. We aimed to review the evidence on transmission of the virus. Real‐world studies have isolated viable viruses from high‐touch surfaces for as long as 15 days. Strong evidence suggests that the current circulating monkeypox (mpox) has evolved from previous outbreaks outside of Africa, but it is yet unknown whether these mutations may lead to an inherently increased infectivity of the virus. Strong evidence also suggests that the main route of current MPXV transmission is sexual; through either close contact or directly, with detection of culturable virus in saliva, nasopharynx, and sperm for prolonged periods and the presence of rashes mainly in genital areas. The milder clinical presentations and the potential presence of presymptomatic transmission in the current circulating variant compared to previous clades, as well as the dominance of spread amongst men who have sex with men (MSMs) suggests that mpox has a developed distinct clinical phenotype that has increased its transmissibility. Increased public awareness of MPXV transmission modalities may lead to earlier detection of the spillover of new cases into other groups.

## 1 INTRODUCTION

The human monkeypox virus (MPXV in short for the virus, mpox for the disease) belongs to the *Orthopoxvirus* genus of the *Poxviridae* family. Orthopoxviruses are large (size range: 140–450 nanometers) viruses with a brick‐like structure and a genome of approximately 200–500 kbp kb that contains over 200 genes.^1^ Major nonhuman hosts of poxviruses include rodents, rabbits and nonhuman primates.^1^, ^2^, ^3^ MPXV was first described in 1958 among monkeys shipped from Singapore to Denmark.^4^ Following this, additional outbreaks were reported in captive monkeys in the United States, the Netherlands, and France.^5^ The first human case of mpox was reported in 1970 in a 9‐month‐old boy from the Democratic Republic of Congo (DRC).^6^

After the first human case, sporadic outbreaks were reported in some countries in west and central Africa, mainly among children in rural, rainforest areas. Several outbreaks subsequently occurred in the DRC with high fatality rates (1%–2%).^7^, ^8^, ^9^, ^10^, ^11^, ^12^, ^13^, ^14^ However, most of these cases were not laboratory confirmed, due to paucity of local diagnostic infrastructure, difficult to reach patients and challenges associated with civil unrest and the existing health system. Until recently, research into mpox has been neglected globally. There have long been concerns that MPXV could over time expand to fill the ecological niche once occupied by the closely related, now eliminated, variola virus.^15^ The combined effects of deforestation, population growth, encroachment on animal reservoir habitats, increasing human movement and enhanced global interconnectedness have made this possibility more real now than ever.^16^, ^17^, ^18^

Mpox reached international attention in 2003, when 71 human cases were reported in the United States.^19^, ^20^, ^21^, ^22^ Between 2003 and 2022, a few travel‐related cases were reported outside endemic countries in Europe, North America, and Asia.^23^, ^24^, ^25^, ^26^, ^27^, ^28^, ^29^, ^30^, ^31^

In May 2022, human mpox cases were reported in Portugal, Spain, Canada, Belgium Sweden, Italy, Australia, France, Germany, the United States, and the UK.^32^ On July 23, 2022, the World Health Organisation (WHO) Director‐General Tedros Adhanom Ghebreyesus declared this outbreak a Public Health Emergence of International Concern.^33^ As of November 16, 2020, a total of over 81 000 confirmed cases in 110 countries have been reported, many of whose had no clear epidemiological links and milder, nonspecific clinical presentations compared to previously circulating virus in Africa.^34^, ^35^ Indeed, cases of mpox infection in the last few months have already exceeded the number of confirmed or suspected human mpox cases over the 20th century. In August 2022, to reduce stigmatization in light of rapidly increasing cases, the WHO renamed the two known clades of MPXV from Congo Basin or Central African clade to clade I and the West African clade to clade II. Subsequent lineages will be named using Roman numerals for the clade and lowercase letters for the subclade.^36^ Most recently on November 28th, to reduce discrimination and stigma that could steer people away from testing and vaccination, WHO announced that “mpox” was now the preferred name for monkeypox (and used throughout the remainder of this manuscript); with both names to be used simultaneously for 1 year while “monkeypox” is phased out.^37^

Successful transmission of any pathogen requires a minimum dose of replication‐competent virus to be delivered to a vulnerable anatomical site in a susceptible host. A combination of viral, host and environmental characteristics affect transmission. We, therefore, undertook a review to assess the empirical evidence relating to the transmission of mpox, in light of the recent surge in a number of cases worldwide. In contrast to existing reviews, we focus on how mpox may have adapted or changed in the recent outbreak to increase its incidence globally.^1^, ^18^, ^38^ Our review can inform rational evidence‐based policies and reduce misinformation on this topic.

## 2 METHODS

We aimed to summarize the major studies that have investigated three main factors that contribute to transmission:

1. Environmental viability of MPXV.
2. Virus and host factors relating to transmission.
3. Population dynamics of the transmission of MPXV.

We manually searched electronic databases (PubMed, EMBASE, and the Cochrane Library) and the *medRxiv* preprint server, for English‐language titles and abstracts published until November 30, 2022; we also searched reference lists of relevant articles and institutional or governmental reports of MPXV transmission. Articles were included if they provided relevant information on the aforementioned factors. Selected articles included laboratory‐based studies of the virus, instructive case and cluster reports, and other observational or modeling studies. In relation to the factors, we focused on studies that could help to explain why there has been rapid, expanding community transmission of MPXV in 2022.

## 3 RESULTS

### 3.1 Environmental viability of the virus

#### 3.1.1 Laboratory studies

Little has been published examining the environmental viability of MPXV under experimental conditions. However, Orthopoxviruses in general remain infectious under dry conditions and different temperatures in the environment for long periods of time. Since 1972, Sparkes and Fenje found dried vaccinia virus to be stable for up to 39 weeks (at 4°C) without loss of infectivity.^39^ Similarly, in 1976, Huq found variola virus to be viable in a low humidity, low‐temperature environment for many years.^40^ Of note, Varreault and colleagues studied the susceptibility to aerosolization of MPXV using a 10.7‐L rotating chamber. Airborne viruses were detected by culture and quantitative polymerase chain reaction (qPCR) after up to 90 hours of aging. Furthermore, the authors found that viral concentrations detected dropped by two logarithms for culture analysis and by one logarithm for qPCR analysis within first 18 hours of aging, but viral concentrations were stable between 19 hours and 90 hours, suggesting a potential for mpox virus to retain infectivity in aerosols for more than 90 hours.^41^

#### 3.1.2 Real‐world settings

In real‐world settings, multiple studies in the United States and UK have identified MPXV DNA from samples taken from contaminated environmental surfaces, most commonly, high‐touch surfaces. Pfeiffer and colleagues assessed the presence and degree of surface contamination of 30 household objects contacted by two mpox patients in Salt Lake City.^42^ 21 (70%) yielded positive real‐time PCR results, however, no specimen yielded a positive viral culture result. In contrast, Nörz et al.^43^ examined surfaces in rooms of hospitals occupied by two mpox patients on their fourth day of hospitalization in Dallas, Texas. Contamination up to 10^5^ viral copies/cm^2^ on inanimate surfaces was estimated by PCR and replication‐competent virus was successfully isolated from surfaces with more than 10^6^ copies/cm^2^. All surfaces directly touched by the patients’ hands showed viral contamination with the highest loads detected in both bathrooms.^43^

Morgan et al.^44^ conducted environmental sampling at the residence of a person in Dallas, Texas USA who had travel‐associated mpox. Targeted environmental swab sampling was conducted 15 days after the person who had mpox left the household. The authors found extensive MPXV DNA contamination in the household; seven samples gave positive viral culture. A significant difference between viable virus detected was found in cultures of porous surfaces, such as bedding and clothing (6/10, 60%) versus nonporous surfaces, such as metal and plastic (1/21, 5%). Viable MPXV was detected on household surfaces for at least 15 days.^44^

Gould et al.^45^ investigated environmental contamination with MPXV from infected patients admitted to isolation rooms in the UK. Surface swabs of high‐touch areas in isolation rooms, of healthcare worker personal protective equipment (PPE) in doffing areas and from air samples collected before and during bedding change were analyzed to assess contamination levels. The authors identified widespread contamination (56 out of 60 samples, MPXV DNA cycle threshold values 24.7–37.4) in occupied patient rooms, on healthcare worker PPE after use and in doffing areas. 5 out of 20 air samples taken were positive; three of four air samples collected during a bed linen change in one patient's room were PCR positive. Replication‐competent virus was identified in two of four samples selected for viral isolation, including from air samples collected during the bed linen change.^45^

### 3.2 Viral factors

#### 3.2.1 Virology

MPXV falls into two distinct clades, based on genetic and geographic variation: formerly Central Africa (or Congo Basin) clade, now known as clade I and formerly the West African clade, now known as clade II. There is approximately 0.5% genomic sequence difference between the two clades, with the former appearing to be more virulent based on higher observed mortality rates.^46^ The genomic differences between clades I and II viruses occur in regions that encode for important virulence genes, and likely contribute to differences in clinically severity.^47^ For example, Hudons and colleagues found that the gene encoding a complement control protein that prevents initiation of the complement pathway is missing in clade II viral strains, and animal models of mpox using the clade I virus with a complement control protein deletion led to reduced morbidity and mortality in prairie dogs.^47^

#### 3.2.2 Mutations in the current outbreak

In the current 2022 outbreak, a new lineage B.1, classified as clade IIb for its close relationship to clade II has been identified.^48^, ^49^ Usually, the double‐stranded DNA of orthopoxiruses is very stable, and their DNA polymerase has proofreading exonuclease acitivity, resulting in a low mutation rate of one to two nucleotide changes per year.^5^ The new B.1 lineage has been associated with strains circulating in Nigeria during the 2017 outbreak, however, the current strain of MPXV in the UK shows 48 single mutations in the genome compared with strain sequencing from 2018.^50^, ^51^ This represents a mutation rate 6–12 times higher than previously estimated.^48^

Sequencing by Isidro and colleagues also found that the circulating MPXV appears to have descended from the clade sampled in cases from Nigeria, Singapore, Israel, and the UK between 2017 and 2019.^52^ Nextstrain, an open‐source project that allows for tracking of pathogen evolution in real time, performed molecular clock analysis of this virus, which showed extensive diversity in MPXV' descendant lineages (A.1, A.2, A.1.1, and B.1).^53^ Within these lineages, O'Toole and Rambaut observed that a cytosine deaminase called apolipoprotein B editing complex (Apolipoprotein B mRNA Editing Catalytic Polypepide‐likee3, APOBEC3) may be driving these rapid mutations.^54^

In the United States, Gigante et al.^55^ identified two lineages of MPXV among two 2021 and seven 2022 mpox cases from the USA, B.1 and a minor contemporaneously sampled variant A.2. Analyses of mutations among these two variants also revealed an extreme preference for GA‐to‐AA mutations indicative of human APOBEC3 cytosine deminase activity; such mutations were also not enriched within other MPXV clades. Indeed, specific enrichment of APOBEC3 motif mutations since 2017 may suggest differences that may have occurred in virus‐host interactions. Since the APOBEC3 human protein serves as a cellular defense mechanism by introducing errors into the viral genome, it is possible that mutations of this type are indicative of a large amount of human‐to‐human transmission. Further research in this area, to explore how these variations affect MPXV transmissibility is urgently required.

### 3.3 Host factors

#### 3.3.1 Transmission from lesions

With regard to the human host, transmission of MPXV was classically thought to be modulated by contact with infectious bodily fluid, including virus present in the generalized monomorphic pustular rashes on the face and hands, with a small degree of aerosol and droplet spread. An infected patient is thought to be noninfectious when their lesions have crusted over.^56^ However, the literature on host factors relating to MPXV transmission, including modes of transmission and duration of infectiousness has also been severely lacking before the current outbreak. A recent PubMed search for “monkeypox AND transmission,” conducted in June 2022 yielded only 224 manuscripts published from 1962 to 2022, with more reviews published on the subject than original research studies in humans.^57^ Only 15 were studies that investigated transmission of MPXV in humans before the current outbreak; the largest study being that of 2510 contacts of 214 patients with mpox in Zaire from 1980 to 1984. Here, investigators reported infected cases without exanthema.^58^

#### 3.3.2 Respiratory transmission

In a retrospective observational study, Adler and colleagues report longitudinal virological findings of seven patients with mpox who were diagnosed in the UK between 2018 and 2021, including the one healthcare worker who acquired the virus nosocomially and the one patient who acquired the virus abroad and transmitted it to an adult and child within their household. In addition to PCR positivity for MPXV DNA in all pleiomorphic skin lesions in these patients (papules, vesicles, pustules, umbilicated pustules, ulcerating lesions, and scabs), the authors found shedding of MPXV DNA in the upper respiratory tract swabs for at least three weeks in three patients, including two that were treated with off‐patent antivirals.^27^

A review of experimental and natural infections of animals with MPXV between 1958 and 2012 found that in nonhuman primates, infection could be initiated by intrabronchial application of 5 × 10^4^ plaque‐forming units (PFU), of Clade I. In respiratory challenge studies with the prairie dog animal model using the Congo Basin Strain, virus titers of 10^4^ and 10^3^ PFU in most cases caused infection, and in one study, 1 out of 4 prairie dogs infected with 6 × 10^2^ PFU MPXV became infected, and showed development of disseminated lesions.^59^ Multiple challenge studies using macaques have shown that delivering an aerosolized MPXV either directly above the tracheal carina via bronchoscope or into a head‐only chamber via a nebulizer are both sufficient for the development of clinical disease that resembles human mpox.^60^, ^61^, ^62^ These findings suggest that whilst transmission is likely to occur from close contact, a degree of transmission could be respiratory related.

#### 3.3.3 Close contact and sexual transmission

One unique feature of the current mpox outbreak appears to be the high prevalence of human‐to‐human transmission following sexual intercourse, either indirectly through close contact, or directly as a sexually transmitted infection. Moreover, the viral load in skin samples from the current outbreak is consistently the highest compared to other body locations, with evidence of replication‐competent virus isolation reported more frequently from skin samples than throat swabs. Eloy José Tarín‐Vicente et al.^63^ studied sexual behavior in relation to clinical presentation and virological outcomes for 181 patients with PCR‐confirmed human MPXV, who were consecutively selected from three sexual health clinics in Spain. Most (92%) identified as MSM; 8% were heterosexual men or women. Mpox infections were linked to previous sexual exposures, with the median number of sexual partners in the past 3 months being 7 (interquartile range [IQR] 3–16). 72 (40%) of participants were HIV positive; 31 (17%) had concurrent sexually transmitted infections and 57 (31%) had used recreational drugs during sex. Types of sexual practices reported were oral insertive sex (160, 88% of participants); oral receptive sex (158, 87%), anal insertive sex (131, 72%), anal receptive sex (108, 60%), and vaginal insertive sex (11, 6%). Compared with other types of sexual practices, anal receptive sex was associated with a higher frequency of viral prodrome (62% vs. 28%, *p* < 0.001) and more frequent proctitis (38% vs. 7%, *p* < 0.001). In contrast to cases within Africa, most cases in the current outbreak are characterized by the presence of ulcerating genital rashes, which precede the development of generalized pustular rashes. High viral loads as well as culturable MPXV have been successfully found in semen samples (see below). All this suggests that the genital area may be a primary site of infection. Sexual and close contact was suspected as a mode of transmission during the 2017 outbreak of human mpox in Nigeria, but this was never proven.^64^

Patel et al.^65^ described 197 patients with mpox who presented to high‐consequence infectious diseases centers in South London between May and July 2022. Compared with cases observed in the Democratic Republic of Congo (DRC), where pox lesions were reported in the head and arms, all patients in this cohort presented with mucocutaneous lesions, most commonly in the genitals (111, 56%), or perianal area (82, 42%). These cases had far fewer lesions than previous outbreaks (mean lesion count at presentation: 370 in the DRC compared with 6 in London). Whilst cases in the DRC reported sore throat, nasal discharge, and congestion as the main symptoms, cases in London presented with rectal pain, sore throat, and penile oedema. Finally, only a quarter of this cohort had known contact with someone with confirmed mpox infection, raising the possibility of asymptomatic (or undetected symptoms) transmission.

Traditionally, patients with poxvirus infections are likely to be infectious from symptom onset (which includes prodromal symptoms before the appearance of the rash) until the lesions scab and fall off, and a new layer of skin has formed.^1^ Peiró‐Mestres et al.^66^ tested 147 clinical samples collected at different time points from 12 patients infected with mpox by real‐time PCR. High viral loads were observed from skin pustules. MPXV DNA was detected in saliva from all cases, sometimes with high viral loads. Other samples that were frequently positive included rectal swabs, nasopharyngeal swabs, semen, urine, and feces. However, the authors did not perform cell culture in these samples. Lapa et al.^67^ investigated viral shedding in longitudinal semen samples collected 5–19 days after symptom onset from one confirmed MPXV case diagnosed in Rome, Italy. MPXV DNA was detected in all semen samples tested during the period of observation. Semen collected on Day 6 after symptom onset was inoculated in Vero E6 cells, with a clear cytopathic effect observed 48 h after the inoculum and MPXV replication was confirmed by real‐time PCR on DNA profiled from cell growth medium collected after 48 h, 72 h, and 96 h. No MPXV DNA was detected in either urine or blood samples, suggesting the absence of semen cross‐contamination from other potential sources.

#### 3.3.4 Transmission between animals and humans

In endemic countries, wild animals (rodents and primates) have been found to carry MPXV.^68^ Animal‐to‐human transmission can occur from noninvasive expsoures to infected animals, such as touching the animal, cleaning its cage, and hunting or processing its meat, or from a bite or scratch from an infected animal. Transmission of MPXV in prairie dogs has also been described in the United States and in captive primates in Europe, that were in contact with imported infected animals.^59^ Recently, Seang and colleagues described a case of a dog with confirmed MPXV infection, acquired through human transmission.^69^ Twelve days after symptom onset, the male Italian greyhound of two men who have sex with men (MSM) with confirmed mpox infection presented with mucocutaenous lesions on its abdomen and anus. These lesions were positive for MPXV DNA, and demonstrated 100% sequence homology to its owners, on the 19.5 kb pairs that were sequenced.

#### 3.3.5 Vertical transmission

Evidence demonstrating vertical transmission of mpox is also emerging. Mbala et al.^70^ reported outcomes of four pregnant women infected with Clade I–2 had miscarriages in the first trimester, and one had fetal death, with the macerated stillborn showing diffuse cutaneous maculopapillary skin lesions involving the head, trunk, and extremities, including the palms of the hands and the soles of feet. Ramnarayan et al.^71^ report a case of neonatal mpox after peripartum transmission within a family cluster in April 2022 in the UK. The infant had an uneventful birth and developed a rash on Day 9 of life.^71^ The rash was initially vesicular, starting on the palms and soles and subsequently spreading to the face and trunk and gradually becoming pustular. PCR testing of the vesicular fluid samples identified MPXV infection; the infant's condition worsened, requiring invasive ventilation a 2‐week course of enteral tecovirimat and intravenous cidofovir. The infant recovered following 4 weeks of intensive care and 14 days of invasive ventilation.

#### 3.3.6 Percutaneous and other routes of transmission

Detectable viral DNA has been found in blood samples amongst infected patients in the UK and United States^27^, ^72^; two case reports have suggested percutaneous transmission following needlestick injuries from supplies used to collect cutaneous lesion samples. Mpox lesions appeared at the site of the needlestick. Transmission of MPXV via the fecal‐oral route has not been documented.^73^

### 3.4 Transmission dynamics of mpox

#### 3.4.1 Estimating the basic reproductive number of mpox in the current outbreak

In infectious disease transmission dynamics, the basic reproductive number, or R_0_ describes the average number of secondary cases generated from an index case in an entirely susceptible population. The R_0_ of mpox has previously estimated to be between 0.57 to a maximum of 1.25.^74^ From the most recent outbreak in western Europe the R_0_ estimates for three study populations in England, Portugal and Spain were all greater than 1, which is the condition for cases to increase, with estimates ranging from 1.40 to 1.80.^75^

Estimates of R_0_ are a function of host susceptibility, as well as the environmental and social factors where the outbreaks occur. Therefore, several factors may elevate R_0_ estimates in the current outbreak, compared to estimates from previous outbreaks in Central and West Africa. First, the transmission of MPXV is likely to have increased over time, due to declining immunity in the population after the end of smallpox vaccination in the general population. Second, direct contact among young MSMs is a source of a significant number of infections in the current outbreak. Therefore the number of onward transmissions per case could be increased due to the setting in which the virus is currently being transmitted (high rates of close contact). Similarly, the number of secondary transmissions per index case can show different levels of heterogeneity. The number of household contacts, knowledge of the condition, and any public health interventions employed will have an impact on these estimates. A systematic review by Beer and Rao in 2019 of previous outbreaks in Central and West Africa found secondary attack rates ranging between 0% and 11% in household contacts that were not vaccinated against smallpox.^38^ Secondary attack rates in household contacts that were vaccinated were much lower, one study conducted in 1988 found a secondary attack rate in unvaccinated household contacts of 9% compared with 1% for vaccinated contacts.^76^ Beer and colleagues also noted that the “crude” secondary attack rates increased between 1981 and 2005 likely reflecting an increase in the proportion of unvaccinated individuals in the population.

#### 3.4.2 Estimating the prevalence of asymptomatic transmission

Traditionally, individuals infected with mpox appear to take a long time to develop symptoms, with a long infectious period.^77^ Schneider and Eichner^78^ calculated that the average generation time of MPXV (consisting of the latent period, plus half the infectious period) to be relatively long (about 20 days); which renders the virus highly vulnerable to interventions. With increased awareness, cases could be detected more rapidly; even if it takes 1 week from symptom onset to detect and isolate cases, the contagious period (15–27 days after the onset of rash) is reduced by over 50%. Assuming lack of asymptomatic/presymptomatic transmission, an R_0_ of 3 would drop to less than 1.5.

However, Ward and colleagues analyzed the transmission dynamics of the current mpox outbreak in the UK with a contact tracing study, linking data on case‐contact pairs and on probable exposure dates.^79^ The study consisted of 2746 people with PCR confirmed MPXV between May 6 and August 1, 2022. The authors investigated the incubation period (time from becoming infected to developing symptoms) and serial interval (the time from symptom onset in a primary case to symptom onset in a secondary case) of a mpox infection using two Bayesian time delay models.

Of particular concern, the authors found that short serial intervals were more common than short incubation periods suggesting considerable presymptomatic transmission, which was validated through linked patient‐level records. For patients who could be linked through personally identifiable data, four days was the maximum time that transmission was detected before symptoms manifested.

Previous research has not found evidence of transmission and substantial shedding of MPXV before symptom onset, which is reflected guidance from WHO and the European Centre for Disease Prevention and Control.^80^, ^81^ Presymptomatic transmission may be indicative of changes to the primary route of transmission in the current international outbreak, where certain types of contact involving high‐intensity interactions will require lower viral loads to transmit. Hence, presymptomatic transmission may just be transmission before symptoms are *detected* rather than true asymptomatic disease, that is, individuals having mpox lesions in areas which they are unaware. Therefore, effective public health messaging must emphasis the importance of looking for lesions in genitalia and other unexpected areas of the body to have any effect on reducing spread.

#### 3.4.3 Heterogeneity of MPXV viral loads between individuals and relation to transmission

There may be considerable heterogeneity in infectious viral load between individuals, the relationship between symptoms and viral load, and the relationship between symptoms and behavior. For example, individuals with large numbers of lesions may be very infectious, but may not be engaging in risk behavior. Modeling studies have investigated some of these heterogeneities and how they affect the short and long‐term dynamics of these outbreaks. For example, Endo and colleagues used a branching process transmission model, fitted to empirically sexual partnership data in the UK and found that there was a heavy‐tailed nature of the sexual partnership distribution, where a small fraction of individuals have disproportionately large numbers of partners can explain the sustained growth of mpox cases among the MSM population, despite the absence of such patterns of spread in past outbreaks.^82^ Brand et al.^83^ investigated the mpox epidemic within the UK population and stimulated control options over a 12‐week projection, using a stochastic descrete‐population transmission model which included MSM status, rate of formation of new sexual partners, and an underlying random‐sized meta‐population structure. They also found that the virus may have already infected a significant proportion of the MSM group with the highest sexual activity (33%; prediction IQR: 16%–45%). The median age of infected individuals in the recent UK outbreak was 37 years, possibly reflecting sexual behavior in the UK. This is in contrast with earlier outbreaks in the DRC where in 2016 the median age was 10 years, and by 2020 only 42% of cases were older than 5 years.^84^, ^85^ They also showed that behavioral change, arising from increased knowledge and health warnings coupled with vaccinia‐based vaccination would decrease the transmission rate of individuals with mpox, leading to case incidence flattening and then declining over the projected 12‐week period.

It is also possible that the current circulating mpox variant represents a similar evolutionary consequence as has been posited for HIV, whereby the reproduction number has been optimized by infected patients having minor symptoms (compared with previous variants in Africa) but remaining highly infectious to close contacts.^86^ This would allow those infected to engage in activities that maximize onward transmission, thus enabling the worldwide spread of the virus. Thornhill and colleagues describe 528 mpox infections between April 27 and June 24, 2022 at 43 sites in 16 countries.^72^ Median age of study participants was 38 years old. 98% of the persons with infection were gay or bisexual men; 75% were White, and 41% had human immunodeficiency virus (HIV). The transmission was suspected to have occurred through sexual activity in 95% of the persons with infection; the median number of sex partners in the previous 3 months was 5; 169 (32%) were known to have visited sex‐on‐site venues within the previous month. 95% of infected persons presented with skin lesions across various parts of the body including the anogenital area; trunk arms or legs; face; and palms and soles. However, only 13% were admitted to the hospital, with only two serious complications (one case of epiglottitis and two cases of myocarditis) reported; both of whom fully recovered. No deaths were reported. Therefore, it is entirely possible that spillover of infection could start to occur in lower‐risk populations other than MSM in the future, as what historically occurred with HIV. Given the strong evidence of fomite transmission, anyone living with infected cases regardless of sexual orientation may be at risk. Nolen and colleagues report the results of a mpox outbreak investigation in the DRC in 2013; they found that the household attack rate (rate of persons living with an infected person that develop symptoms of mpox infection) to be 50%, with a mean incubation period of 8 days.^85^ Increased public awareness of MPXV transmission modalities may lead to earlier detection of new cases into other groups, as well as timely vaccination of risk groups and individuals who are immunocompromised. Indeed, clinical features of mpox in women and nonbinary individuals appear to be similar to those described in men.^87^

## 4 CONCLUSIONS

**Figure 1:**
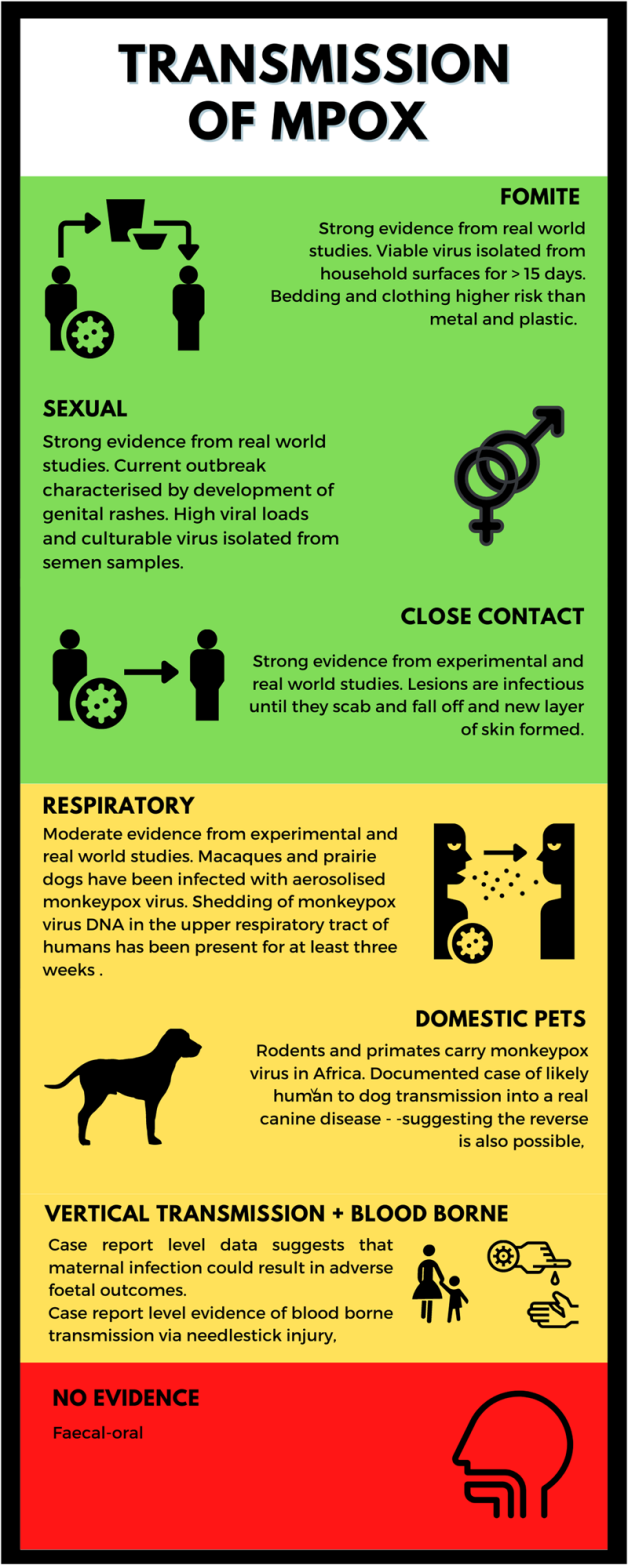
Infographic illustrating the various routes of mpox transmission and strength of evidence.

Before the current outbreak, relatively little evidence exists regarding the transmission dynamics of MPXV. A summary of our appraisal of the evidence regarding various modes of transmission can be found in Figure 1, where green indicates strong evidence, amber moderate evidence, and red: little to no evidence. Current circulating strains of MPXV worldwide are environmentally stable. Viral DNA and culturable virus have also been isolated from patients with mpox, in the various sites of their rash as well as within the nasopharynx, groin, anogenital areas, sperm, and contaminated materials such as bedding. Together these highlight the potential of fomite, close contact, aerosol and sexual transmission. Further research into the modes of transmission is urgently needed as this will have an impact on the interventions required to prevent further mpox infections. Given the potential for aerosol transmission and the number of reports of outbreaks in healthcare settings the use of appropriate PPE is essential to prevent nosocomial transmission. Current WHO guidance includes the use of a respirator for healthcare works when providing care for patients with mpox.^88^

Previous estimates of the R_0_ of mpox are likely to have underestimated the current outbreak, probably because they were in populations with much less close contact. Whether mutations that are driving the current variants of mpox circulating worldwide also contribute to increased infectivity remains unknown. However, compared to previous variants, most patients infected with the current variant are MSMs, engage in high‐risk sexual activity, and remain systemically well before, during, and after their infection, with significantly fewer pox lesions compared to previous clades and focused in the groin areas. This suggests that the current MPXV variants have adapted (both genetically, and as a result of human behavior) to maximize the probability of onward transmission. Increased public awareness of MPXV transmission modalities may lead to earlier detection of the spillover of new cases into other groups.

## AUTHOR CONTRIBUTIONS

Daniel Pan and Julian W. Tang conceived the idea of the manuscript. Daniel Pan and Shirley Sze wrote the first draft of the manuscript. Christopher A. Martin, Joshua Nazareth, Christopher A. Martin, Jonathan Decker, Eve Fletcher, T. Déirdre Hollingsworth, Michael R. Barer, Manish Pareek, and Julian W. Tang commented and edited subsequent versions of the manuscript. All authors have reviewed and approved the final version of the manuscript submitted to the journal.

## CONFLICTS OF INTEREST STATEMENT

The authors declare no conflicts of interest.

## Data Availability

Data sharing is not applicable to this article as no datasets were generated or analyzed during the current study.
